# Does Characterization of Phenotypic Heterogeneity Provide Mechanistic Insights or Influence the Response to Treatment? Experience in Positive and Neutral Trials in Patients With Heart Failure and a Preserved Ejection Fraction

**DOI:** 10.1161/CIRCULATIONAHA.126.079235

**Published:** 2026-07-21

**Authors:** Milton Packer, Gabriele G. Schiattarella, Mark C. Petrie, Daniel Burkhoff, Javed Butler, Carolyn S.P. Lam, Faiez Zannad, Muthiah Vaduganathan, Barry A. Borlaug

**Affiliations:** Baylor Heart and Vascular Institute, Dallas, TX (M.P.).; Imperial College, London, UK (M.P.).; Max Rubner Center for Cardiovascular Metabolic Renal Research (MRC), Deutsches Herzzentrum der Charité (DHZC), Charité-Universitätsmedizin Berlin, Germany (G.G.S.).; Division of Cardiology, Department of Advanced Biomedical Sciences, Federico II University, Naples, Italy (G.G.S.).; School of Cardiovascular and Metabolic Health, University of Glasgow, UK (M.C.P.).; Cardiovascular Research Foundation, New York, NY (D.B.).; Baylor Scott & White Research Institute, Dallas, TX (J.B.).; University of Mississippi School of Medicine, Jackson (J.B.).; Cardiovascular and Metabolic Disorders Programme, Duke–National University of Singapore Medical School (C.S.P.L.).; National Heart Centre Singapore (C.S.P.L.).; Université de Lorraine, Centres d’Investigation Clinique, INSERM, Centre Hospitalier Régional Universitaire de Nancy, France (F.Z.).; Division of Cardiovascular Medicine Brigham and Women’s Hospital, Harvard Medical School, Boston, MA (M.V.).; Department of Cardiovascular Medicine, Mayo Clinic, Rochester, MN (B.A.B.).

**Keywords:** adipokines, adiposity, cluster analysis, genetic heterogeneity, heart failure with preserved ejection fraction, mechanistic heterogeneity, phenotypic heterogeneity

## Abstract

At a time when there was no unifying hypothesis and no broadly effective treatments for heart failure with a preserved ejection fraction (HFpEF), it was proposed that HFpEF represented a multitude of different disorders. Accordingly, characterization of phenotypic heterogeneity was envisioned as a means of identifying novel causal pathways that could lead to new treatments while simultaneously discerning patients who would selectively benefit from a specific therapy. However, efforts over the past decade to characterize phenotypic diversity in diverse and complex ways have not achieved these goals. Subgroup analyses of neutral HFpEF trials have failed to reliably identify responders to treatments. Neither proteomics nor unsupervised cluster analysis across multiple phenotypic domains has yielded reproducible results (even in the same dataset), elucidated novel mechanistic pathways for new drug development, or identified patients most likely to benefit from treatment. In marked contrast to these efforts to characterize phenotypic heterogeneity, large-scale trials in HFpEF enrolled patients using broad eligibility criteria, and they established the benefits of sodium-glucose cotransporter 2 inhibitors and mineralocorticoid receptor antagonists without evidence for subgroup effects. However, it is noteworthy that patients enrolled in recent large-scale HFpEF trials were characterized by general uniformity with respect to the presence of excess adiposity, with central obesity being a feature of the vast majority of enrolled participants. Of note, patients with obesity showed particularly large benefits when treated with effective drugs for HFpEF. These observations raise the possibility that, if these trials had permitted the participation of patients with class III obesity or with lower natriuretic peptide levels, the magnitude of the observed treatment effects might have been greater than originally reported. These findings are consistent with a central role for visceral adiposity (and the secretion of an altered suite of adipokines) in the pathogenesis of HFpEF. Therefore, because the field of HFpEF has a unifying hypothesis (applicable to the large majority of patients), enrolled a broad population of patients in large-scale trials without subgroup interactions, and has several broadly applicable effective treatments (as is true for heart failure with a reduced ejection fraction), the motivation to characterize phenotypic heterogeneity in HFpEF in complex ways may no longer be supported or needed.

Heart failure with a reduced ejection fraction (HFrEF) can exhibit a broad range of phenotypic expressions, including the variable prominence of diverse pathophysiological features (eg, fluid retention, left ventricular enlargement, myocardial ischemia, chronic kidney disease, skeletal muscle abnormalities, pulmonary hypertension, natriuretic peptides). However, despite phenotypic heterogeneity, there is a unifying hypothesis to explain the evolution and progression of HFrEF (eg, neurohormonal derangements),^1^ and in general, the central pathophysiological importance of neurohormonal activation does not depend on the cause of the myocardial disorder. Furthermore, large-scale trials in HFrEF enrolled a broad population of patients and did not focus on phenotypic differences, and physicians treat patients with HFrEF with a similar suite of foundational drugs, regardless of the cause of cardiomyopathy (genetic, ischemic, etc). Although patients with HFrEF can exhibit considerable differences in the symptom severity, hemodynamic features, and comorbidities, they respond similarly to treatment with foundational drugs for HFrEF, regardless of whether they have coronary artery disease, diabetes, or hypertension or have meaningful right ventricular involvement or impairment of kidney function. Furthermore, the measurement of circulating neurohormonal factors was not an entry criterion for participation in HFrEF trials, and it is not performed in clinical practice to select patients for treatment with neurohormonal antagonists. These observations, taken together, support the conclusion that characterization of phenotypic heterogeneity has not advanced our understanding of the mechanisms underlying or the treatment of HFrEF.

For the past 10 to 15 years, it has been believed that, in contrast to HFrEF, phenotypic heterogeneity is more pathophysiologically important in heart failure with a preserved ejection fraction (HFpEF). How did this belief arise? Can characterization of phenotypic heterogeneity provide important mechanistic insights into and treatment guidance for HFpEF when it has not done so in HFrEF?

For purposes of this article, we define HFpEF as symptoms and signs of heart failure with a left ventricular ejection fraction of ≥50% after the exclusion of HFpEF mimics, for example, cardiac amyloidosis, hypertrophic and infiltrative cardiomyopathies, valvular and pericardial disease, and end-stage kidney disease. These separate disease entities were well recognized before HFpEF was defined as a clinical entity; they have distinct features on blood testing or cardiac imaging that allow a diagnosis during an initial clinical evaluation; and they have been excluded from participation in randomized controlled trials of new interventions for HFpEF. Similarly, we define treatments for HFpEF as interventions that have been tested in randomized controlled trials that focused on the HFpEF disease process, in contrast to the general care of patients with other cardiovascular conditions (eg, hypertension, atrial fibrillation, and mitral regurgitation).

## Goals of Characterizing Phenotypic Heterogeneity in Heart Failure

**Figure 1.**
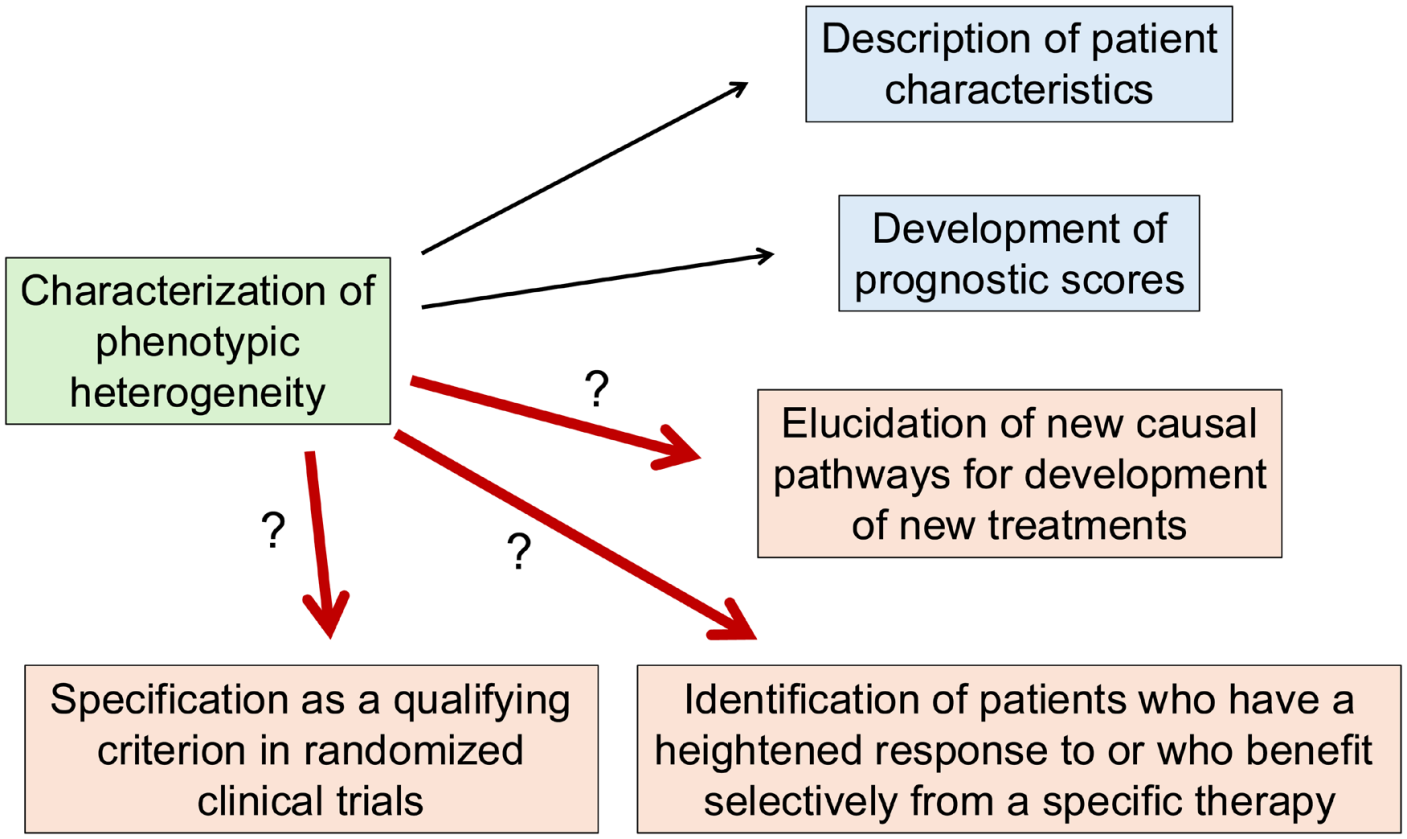
**Potential goals of characterizing phenotypic heterogeneity.** Although baseline characteristics of patients can be used to describe clinical features and to develop prognostic scores, the most important aspirational goals of characterizing phenotypic heterogeneity are (1) to identify patients with a heightened or attenuated response to (or who show selective benefit from or fail to respond favorably to) current broad-based treatments; (2) to designate the features of the phenotype as a qualifying criterion for enrichment of participation in new randomized controlled clinical trials, particularly to enhance the likelihood of discerning a treatment effect; and (3) to elucidate mechanistic evidence for the identification of causal pathways for recognition of new disease states or the development of novel treatments. These principal goals are shown in red arrows, but thus far, characterization of phenotypic heterogeneity has not fulfilled these 3 important goals.

There are 2 broad reasons why physicians might seek to characterize phenotypic heterogeneity in patients with heart failure (Figure 1). First, physicians may seek to describe the diversity of phenotypic features because specific clinical, hemodynamic, and pathophysiological characteristics may portend a favorable or poor prognosis. It is well known that patients with advanced disease (as reflected by functional capacity or circulating levels of natriuretic peptides) fare worse than those who present at an earlier stage in their illness and that patients with ≥1 serious comorbidities (eg, diabetes, chronic kidney disease, pulmonary hypertension) have a particularly heightened risk of death or hospitalization. It is possible to combine these features to develop prognostic scores,^2^ but these scores are rarely used in the care of patients with heart failure or for the recruitment of patients into clinical trials. Furthermore, although these prognostic scores may reflect the burden of disease, they do not identify causal or actionable mechanisms that promote the evolution or progression of the underlying disorder. In fact, despite the importance of neurohormonal mechanisms in HFrEF, circulating levels of neurohormonal factors are not included in prognostic scores, and physicians do not use neurohormonal measurements to determine whether HFrEF should be treated with specific drugs.

Second and far more important, physicians may seek to characterize phenotypic heterogeneity in the hope that the precise and thoughtful assessment of clinical or pathophysiological features might identify novel causal pathways that could define new diseases and lead to new treatments while simultaneously discerning which patients might benefit selectively from a specific therapy (Figure 1). If patients with heart failure who have diabetes were to consistently show exaggerated benefits from drugs that lower blood glucose, this finding would help to tailor treatments to enhance the probability of a favorable treatment effect. More important, such a finding would also provide critically important mechanistic insights, specifically supporting the hypothesis that hyperglycemia (per se) was playing a key role in promoting the progression of heart failure. Furthermore, if a clinical feature such as diabetes were able to predict a particularly favorable treatment response, it could be prospectively designated as a qualifying criterion in the design of randomized clinical trials of patients with heart failure.

**Figure 2.**
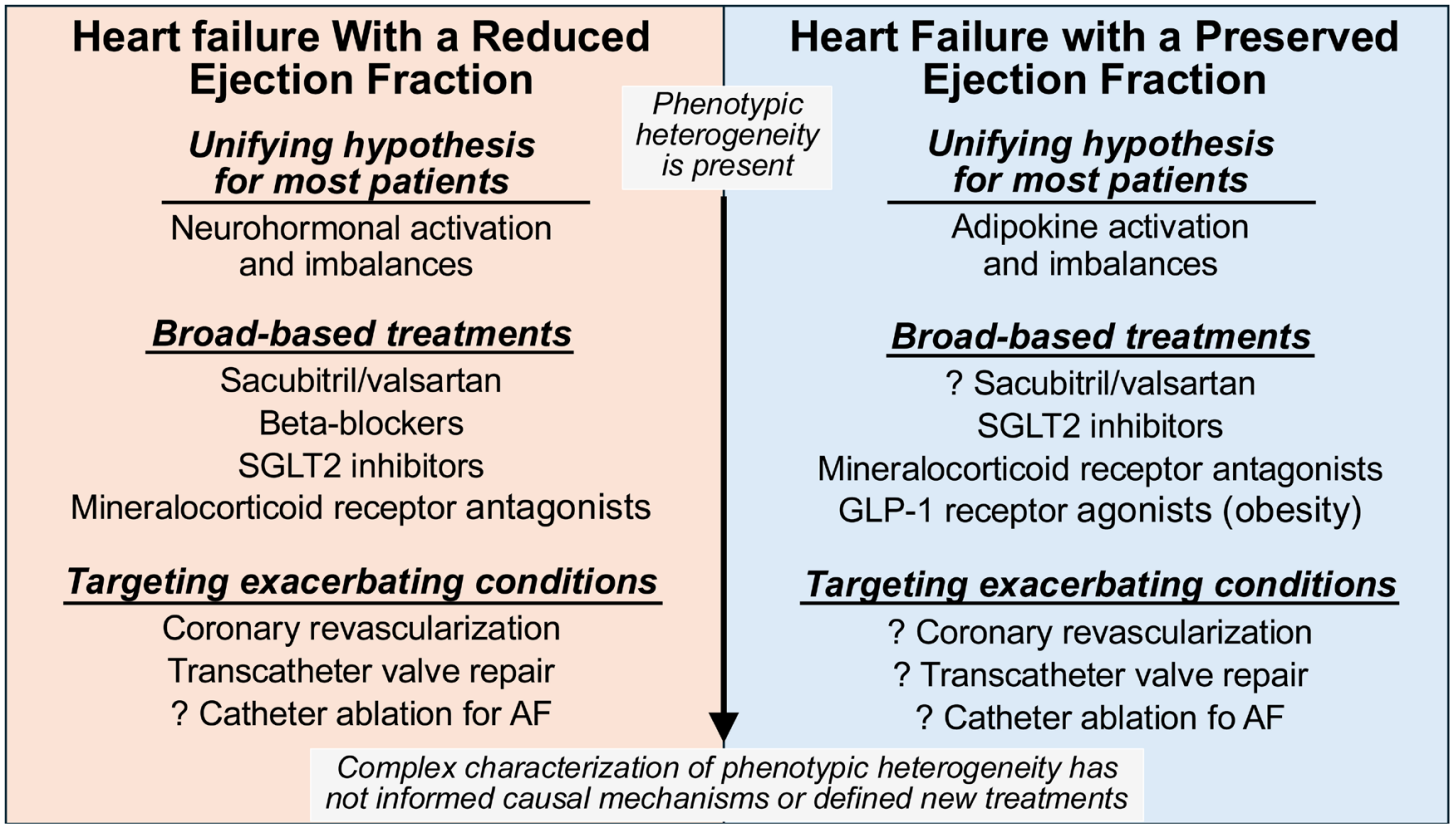
**Characterization of phenotypic heterogeneity in patients with HFrEF or HFpEF.** Although phenotypic diversity is a ubiquitous feature of patients with heart failure and either a reduced or preserved ejection fraction (HFrEF and HFpEF, respectively), complex characterization of phenotypic heterogeneity has not discerned novel causal mechanisms, identified targets for new drugs, defined the responses to treatment, or guided the use of effective drugs in clinical practice. At the present time, both HFrEF and HFpEF are characterized by a unifying hypothesis (which applies to the large majority of patients) and by effective broad-based treatments (which are not prescribed selectively in subgroups defined by baseline characteristics or phenogroups). In both HFrEF and HFpEF, physicians also treat exacerbating conditions (eg, coronary artery disease, mitral regurgitation, atrial fibrillation [AF]), which are used in concert with drugs that target neurohormonal or adipokine imbalances, respectively. Question marks are placed to denote uncertainty in the presence of a treatment benefit, whereas HFpEF is defined as ejection fraction of ≥50% and the absence of HFpEF mimics. Glucagon-like peptide 1 (GLP-1) receptor agonists have been evaluated to date only in patients with HFpEF with a body mass index of ≥30 kg/m^2^ but have been shown to prevent HFpEF in patients with a body mass index of ≥27 kg/m^2^. SGLT2 indicates sodium-glucose cotransporter 2.

Therefore, the principal aspirational goal of phenotypic characterization is not to presage disease severity or prognosis but to guide the development and optimal use of effective drug treatments by (1) distinguishing responders from nonresponders to established therapies; (2) identifying specific features that function as qualifying criteria for participation in clinical trials to enhance the magnitude of the treatment effect; (3) elucidating the novel causal mechanisms that promote the evolution and progression of HFpEF and serve as targets for new drug development; and (4) potentially identifying new HFpEF mimic diseases. These goals would seem to be particularly attractive in HFpEF because until recently the field of HFpEF has lacked a unifying mechanistic framework or broad-based effective treatments (Figure 2).

### Evolution of a Belief in Exceptional Mechanistic Heterogeneity in HFpEF

In response to the apparent lack of progress in our approach to HFpEF, Shah et al^3^ proposed in 2015 that disappointments in understanding and treating HFpEF might be explained by its exceptional phenotypic heterogeneity. In doing so, it was assumed that phenotypic heterogeneity implied and could inform mechanistic heterogeneity, even though it was well known that the two were not related. Nevertheless, it was postulated that HFpEF was actually a multitude of different disorders that shared a convergent clinical presentation.^3^ If this conceptualization were valid, efforts to delineate well-defined subphenotypes under the umbrella of HFpEF might reveal insights about different causal pathways and allow personalized treatment of individual patients.

However, despite the proposal for exceptional phenotypic and mechanistic heterogeneity in HFpEF, large-scale clinical trials continued to recruit patients using broad eligibility criteria,^4–6^ and they did not generally focus on elucidating the influence of any particular phenotype. Although sodium-glucose cotransporter 2 (SGLT2) inhibitors and glucagon-like peptide 1 (GLP-1) receptor agonists were initially developed as antihyperglycemic agents, trials of these drugs in HFpEF included patients with and without diabetes.^5,7^ Similarly, although angiotensin receptor neprilysin inhibitors and mineralocorticoid receptor antagonists were effective in lowering blood pressure, trials of these drugs in HFpEF included patients with and without hypertension.^4,6^ The broad scope of patient enrollment in these large-scale clinical trials was conceptually inconsistent with an embrace of mechanistic heterogeneity, but it represented the ideal substrate for testing the potential for mechanistic heterogeneity, which could be evaluated by subgroup analysis based on clinical, pathophysiological, and biomarker measurements performed before the start of randomized treatment.

### Replicability of Subgroup Findings in Heart Failure Trials

However, interpretation of subgroups in a clinical trial must be performed with caution. In general, it is possible to have a modicum of confidence in a subgroup finding if 2 conditions are met: (1) the trial demonstrated a treatment effect in the overall randomized patient population and (2) an outsized effect or lack of a benefit observed in 1 specific subgroup could be consistently replicated in subsequent trials with the same (or similar) treatments in the same disease state. However, history has shown that, when a trial has not observed an overall treatment effect, subgroup analyses (often carried out in the hope of finding some encouraging post hoc signal) are poorly positioned to identify a group of responders or nonresponders who are likely to be validated in a subsequent trial. Differences in the direction and magnitude of a treatment effect in subgroups (even when prespecified and when based on a variable used to stratify randomization) in neutral trials are typically not replicated.^8^

The lack of replicability of subgroup findings is well illustrated by the experience in the PRAISE trial program (Prospective Randomized Amlodipine Survival Evaluation), which evaluated the effect of amlodipine in patients with HFrEF. In the PRAISE-1 trial,^9^ randomization was stratified by the presence or absence of coronary artery disease as the cause of cardiomyopathy, and this stratification variable was the primary prespecified subgroup of interest. Overall, the trial showed that long-term treatment with amlodipine did not reduce morbidity and mortality in patients with HFrEF. The hazard ratio for the effect of amlodipine on the composite primary end point of all-cause mortality and cardiovascular hospitalization was 0.91 (95% CI, 0.86–1.10; *P*=0.31). However, when the results were analyzed according to the main stratification variable of the trial, amlodipine exerted no benefit in patients with ischemic heart disease, but the drug reduced the risk of death by 46% (*P*<0.001) in patients with nonischemic cardiomyopathy (treatment-by-subgroup interaction *P*=0.004).

Because the outcome measure was clinically important, the subgroup was the basis of the stratified randomization of the trial and the *P*_interaction_ value was highly significant, it seemed possible that the subgroup finding might be valid and replicable. Belief in the subgroup finding was even linked to an imagined mechanism: Patients with a nonischemic dilated cardiomyopathy might have coronary microvascular spasm that might be particularly responsive to a calcium antagonist. However, when the efficacy of amlodipine was retested in the subsequent large-scale confirmatory PRAISE-2 trial,^10^ the drug did not reduce all-cause mortality in patients with a nonischemic cardiomyopathy (hazard ratio, 1.09 [95% CI, 0.92–1.29]; *P*=0.33), although the second trial was performed with the same trial protocol, the same dose of amlodipine, and the same group of investigators. The experience in the PRAISE trial program reinforces the need for skepticism in interpreting and embracing the results of subgroup analyses in randomized controlled trials of patients with heart failure.

## Meaningfulness of Phenotypic Heterogeneity in HFpEF Trials That Did Not Demonstrate a Treatment Effect in the Overall Population

The lack of reproducibility of prespecified and statistically significant subgroup interactions in randomized controlled trials of drugs for HFpEF is also well established. In the PEP-CHF trial (Perindopril in Elderly People With Chronic Heart Failure), perindopril exerted no significant effect on the primary end point in patients with HFpEF, but in a prespecified subgroup analysis, a history of myocardial infarction appeared to influence the magnitude of the treatment effect (*P*_interaction_=0.034). Patients with a prior myocardial infarction exhibited a 62% risk reduction, whereas there was little effect in patients without such a history.^11^ However, subsequent trials with inhibitors of the renin-angiotensin system in HFpEF did not confirm this reported subgroup finding.^12,13^ In the INDIE-HFpEF trial (Inorganic Nitrite Delivery to Improve Exercise Capacity in HFpEF), treatment with inorganic nitrite did not improve peak exercise capacity in patients with HFpEF, but there were statistically significant interactions based on 2 prespecified variables—natriuretic peptide levels and atrial fibrillation (*P*_interaction_=0.04 and 0.02, respectively)—with benefits seen in patients with higher natriuretic peptide levels and those with a history of atrial fibrillation.^14^ However, these subgroup effects were not noted in subsequent HFpEF trials with other nitrites and nitrates.^15,16^ Last, in the CAPACITY-HFpEF trial, praliciguat did not improve exercise performance in the trial overall, but there were statistically significant interactions based on 2 prespecified subgroups—age and sex (*P*_interaction_=0.004 and 0.04, respectively)—with benefits seen in younger patients and in women.^17^ However, these subgroup findings were not confirmed in trials with another soluble guanylate cyclase stimulator, vericiguat, in patients with HFpEF.^18,19^

### The Importance of Actionable and Intervenable Phenotypes: The Example of Pulmonary Hypertension and Pulmonary Vascular Disease as a Tentative HFpEF Mechanism

For a phenotype to have mechanistic significance, it should reflect an identifiable pathophysiological causal pathway, the amelioration of which by a specific treatment produces demonstrable clinical benefits in randomized controlled clinical trials. In science, the critical distinction between correlation and causation requires demonstration that modulation of the proposed mechanism has the predicted downstream effect. It is therefore noteworthy that pulmonary hypertension was one of the earliest phenotypic subgroups of HFpEF that was proposed for specific pharmacological targeting in the hope that selective alleviation of elevated pulmonary artery pressures might produce symptomatic and prognostic benefits in HFpEF.^3^

As HFpEF evolves and left atrial hypertension ensues, ≈80% of patients exhibit an elevation of pulmonary artery pressures at rest or during exercise.^20^ In ≈30% of patients, pulmonary vascular resistance is increased at rest or at peak exertion, a finding that is believed to reflect adverse structural remodeling of the pulmonary vasculature.^21^ The impediment to the transit of blood flow across the lungs is often accompanied by right ventricular dilatation and dysfunction and often functional tricuspid regurgitation, leading to worse aerobic capacity and a poor prognosis. Given the potential pathophysiological importance of these pulmonary vascular abnormalities, physicians hoped that drugs that acted as pulmonary vasodilators to selectively reduce pulmonary vascular resistance would decompress right-sided heart pressures and produce an exaggerated benefit in patients with HFpEF, particularly those with an elevated pulmonary vascular resistance.

Despite this expectation, numerous classes of drugs that cause pulmonary vasodilation either by enhancing nitric oxide–cGMP signaling (eg, nitrates, inorganic nitrites, soluble guanylate cyclase stimulators, and phosphodiesterase 5 inhibitors) or by modulating other pathways (eg, relaxin receptor agonists or endothelin antagonists) have not been effective in the treatment of HFpEF (Table 1),^14–19,22–29^ although many of these agents have established clinical benefits in patients with pulmonary arterial hypertension and increased pulmonary vascular resistance who do not have elevated left-sided pressures. It is important to note that, in several HFpEF trials, these pulmonary vasodilator drugs acted to impair activities of daily living and were accompanied by an increased risk of pulmonary congestion, adverse cardiovascular events, and all-cause mortality,^22,25,27–29^ presumably because a drug-related pulmonary vasodilator effect would act to enhance the transit of blood flow across the lungs, leading to increased left atrial pressures in the setting when these pressures are already abnormally elevated as a result of HFpEF. Treatment with pulmonary vasodilators has not been effective (and may have been harmful) even in patients with HFpEF who had established heightened pulmonary vascular resistance, leading to combined precapillary and postcapillary pulmonary hypertension.^25,27^

**Table 1.**
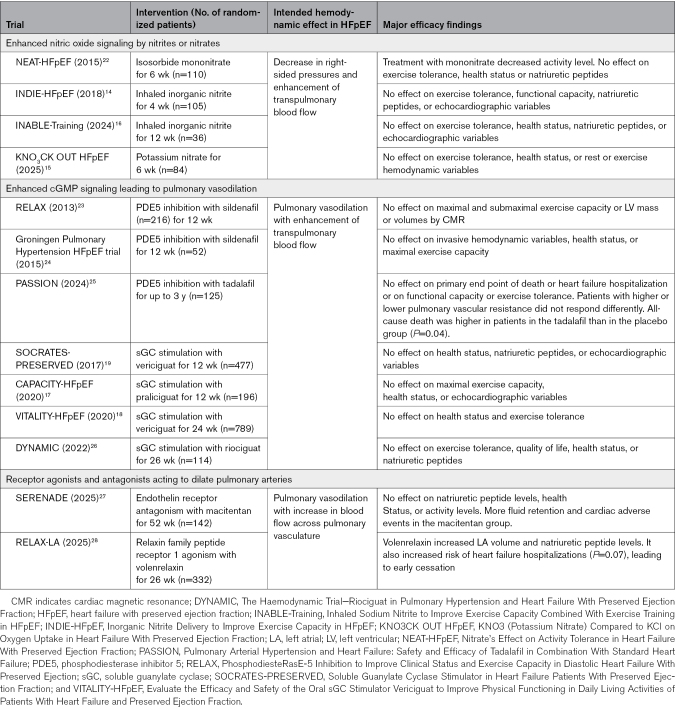
Trials of Drugs With Pulmonary Vasodilator Effects in Patients With HFpEF

Therefore, although pulmonary hypertension represents a distinct hemodynamic derangement with prognostic implications, clinical trials have not validated pulmonary hypertension as a mechanistically actionable and therapeutically meaningful phenotype in patients with HFpEF. In fact, structural changes in the pulmonary arterioles have canonically been considered to exert a protective effect to ameliorate the development of pulmonary congestion,^10,21^ although pulmonary venous remodeling may represent a dominant response to left atrial hypertension. Patients with HFpEF and pulmonary vascular disease display greater lung congestion for any degree of left atrial hypertension, consistent with additional pulmonary capillary pressurization as a result of pulmonary venous disease.^21^ Accordingly, although drugs that dilate pulmonary arterioles (eg, bosentan, macitentan, epoprostenol, sildenafil, tadalafil, and riociguat) produce clinical benefits in patients with pulmonary arterial hypertension without HFpEF, these drugs are not effective and may exacerbate heart failure in patients with left-sided heart failure (by exacerbating pulmonary capillary hypertension), unless they simultaneously act to exert an independent effect to improve the structure and function of the left side of the heart.^26,30,31^

Current evidence suggests that a primary driver of the development of HFpEF, visceral adiposity and adipokine imbalances,^32^ is also a major mechanism promoting the development of pulmonary vasculopathy. The presence of obesity and central adiposity is strongly linked to the development of pulmonary hypertension,^33^ and the adipokine imbalances seen in patients with HFpEF and visceral adiposity promote adverse structural changes in the pulmonary vascular system in experimental models of nutrient excess.^34^ Conversely, GLP-1 receptor agonists and SGLT2 inhibitors ameliorate visceral adiposity,^32^ attenuate the development of experimental pulmonary vasculopathy, and lower pulmonary artery pressures in HFpEF.^35,36^ In addition, activin A, an adipokine secreted by visceral adipose tissue that exerts direct adverse cardiovascular effects,^37^ signals through activin type II receptors and has been directly implicated in the development of pulmonary arterial hypertension and HFpEF.^38^ Activin type II receptor traps (eg, sotatercept) have focused on patients with elevated pulmonary vascular resistance,^39^ but they may be effective in lowering left-sided pressures in patients with HFpEF independently of their effect on pulmonary vascular disease.^31^

### The Pursuit of Mechanistic Heterogeneity in Trials of Devices That Modulate Right Ventricular Filling

Complex physiological constructs have been used to form the basis of subgroup analyses in trials of devices that modulate right ventricular filling in patients with HFpEF. The REBALANCE-HF pilot trial evaluated the effect of endovascular splanchnic denervation in patients with HFpEF,^40^ an intervention that was designed to limit the shift of blood from the splanchnic reservoir to the central circulation during exercise, with the hope of lowering exertional right- and left-sided ventricular filling pressure pressures.

Overall, endovascular splanchnic denervation in the REBALANCE-HF pilot trial did not produce hemodynamic or clinical benefits in the overall population of patients with HFpEF.^40^ However, a post hoc analysis that combined information on the early diastolic flow dynamics, orthostatic changes in pulse pressure, and exercise-induced changes in cardiac output was used to construct a post hoc *z* score composite that defined a favorable response based on a weighting that combined changes in pulmonary wedge pressure at 1 month and changes in health status, 6-minute walk distance, and natriuretic peptides at 6 months.^40^ The investigators envisioned that a favorable response to the intervention might have been seen in patients who had an ability to maintain or augment cardiac output with either exercise or the transition from supine to standing.^40^ Despite these efforts, patients who were classified as responders still did not show significant benefit after endovascular splanchnic denervation on the primary end point of the trial. Furthermore, patients with HFpEF who had echocardiographic evidence of diastolic dysfunction were least likely to respond favorably to treatment.^40^ The validity of this post hoc subgroup analysis is being tested in a new trial.

Complex subgroups have also been used to rationalize the neutral results with other device interventions in HFpEF. Placement of an intra-atrial shunt in HFpEF is predicated on the expectation that the procedure might alleviate the symptoms attributable to exercise-induced left atrial hypertension. However, it is not clear that pulmonary capillary wedge pressures during exercise predictably contribute to effort-related dyspnea.^41^ Furthermore, insertion of the shunt in patients with HFpEF recirculates blood to the right side of the heart, causing right ventricular enlargement^42^ and potentially impairing left ventricular filling because of ventricular interdependence. In REDUCE-LAP II (Study to Reduce Elevated Left Atrial Pressure in Patients With Heart Failure II) and the RELIEVE-HF (Reducing Lung Congestion Symptoms Using the v-Wave Shunt in Advanced Heart Failure), patients with HFpEF treated with an intra-atrial shunt did not benefit or had a higher risk of worsening heart failure outcomes than their untreated counterparts.^43,44^

Potentially motivated to discern a positive signal among the disappointing results of the REDUCE-LAP II trial, the investigators carried out post hoc subgroup analyses, leading to the hypothesis that patients with a peak exercise pulmonary vascular resistance <1.74 Wood units might have experienced a more favorable clinical response.^45^ These observations were predicated on the thesis that an adaptable pulmonary vascular system might benefit from mechanical efforts to increase blood flow to the right side of the heart. However, this hypothesis was based on a post hoc subgroup finding centered on an arbitrary value for exercise pulmonary vascular resistance, defined by the highest tertile among participants in this trial. Pulmonary vascular resistance is subject to considerable measurement variability, particularly during peak exercise, and it is difficult to measure reproducibly, even at specialized centers. Furthermore, pulmonary vascular resistance is an arbitrary ratio that is not a linear reflection of total arterial compliance, particularly when left-sided pressures are elevated.^46^ The validity of the proposed threshold could not be tested in the RELIEVE-HF trial, which did not measure pulmonary vascular resistance during exercise. The RESPONDER-HF trial is now being carried out to evaluate the potential REDUCE-LAP II subgroup results.

### Proteomics as a Tool to Characterize Potential Mechanistic Heterogeneity in HFpEF

Some investigators have sought to characterize complex subgroups and to identify mechanistic pathways among patients with HFpEF based on proteomic measurements performed in circulating blood. Current methods allow the simultaneous estimation of thousands of proteins in tiny quantities of blood, with many based on the use of high-throughput, affinity-based technologies that vary in their binding chemistries, yielding relative values rather than absolute concentrations. Some have hoped that relative protein levels may point to biologically relevant pathways; however, discernment and characterization of causal pathways are exceptionally difficult because the source of these proteins in the bloodstream is not known and because the results of proteomic analyses in heart failure have not yielded concordant results across studies. Furthermore, the ontological grouping of these proteins has thus far been based on their functional characterization in the genesis of cancer rather than cardiovascular disease.

Proteomic analyses in patients with HFpEF have identified an exceptional diversity of differentially expressed proteins.^47,48^ Reports have noted inconsistent associations of some proteins with certain clinical or pathophysiological features of the disease, demonstrating variable linkage to a poor prognosis.^49–51^ Proteomics analyses have generally been interpreted as being concordant with signaling through pathways already known to represent features of experimental HFpEF (adipokine upregulation, inflammation, hypertrophy, fibrosis, and derangements in cell metabolism)^47,49,50,52–54^ or are consistent with mechanisms already postulated to mediate the effects of spironolactone or empagliflozin.^55,56^ However, no clustering of proteins has distinguished any subgroup of patients with HFpEF who are more or less likely to respond favorably to any specific treatment for HFpEF or has identified any new causal pathway that might lead to the development of a novel drug. Proteomic analyses of myocardial tissue (in contrast with the measurement of blood levels) from patients with HFpEF are sparse.^57,58^

### Use of Deep Phenotyping, Cluster Analysis and Phenomapping to Characterize Mechanistic Heterogeneity in HFpEF

In the mid-2010s, at a time when there were no broad-based effective treatments and no unifying hypothesis for HFpEF, it was proposed that HFpEF might represent the end result of many different disorders driven by distinct and independent causal mechanisms.^3^ The proposal that HFpEF was mechanistically exceptionally heterogeneous provided a comforting explanation in a field confused and plagued by the disappointing results from several large-scale trials. Accordingly, some investigators hoped that detailed characterization of phenotypes (or clusters of phenotypic features) might provide unique insights into underlying mechanisms, although it is well known that phenotypic diversity does not imply or inform mechanistic diversity.

Some investigators pursued the possibility that combinations of hundreds or thousands of variables measured across multiple dimensions (ie, clinical features, invasive hemodynamics, assessment of multiple organ functions, and noninvasive imaging of diverse tissues, coupled with the measurement of proteins, bioactive lipids, and metabolites in the circulating blood or on biopsied tissue) might define relevant subgroups among patients with HFpEF, which might point to unique pathways or determine the response to treatment.^3^ The enormous datasets assembled during the course of “deep phenotyping” would be analyzed to seek “patterns” in the data, typically using mathematical approaches such as machine learning (specifically cluster analysis) to perform “phenomapping.”

Unsupervised cluster analysis uses methods ranging from algorithmic (hierarchical or nonhierarchical) clustering to latent class analysis. As usually performed, cluster analysis requires the researcher to make subjective decisions in the selection of measures that would define how similar items need to be to justify the formation of a cluster. Different clustering algorithms have different optimization functions, which include specification of the number of clusters or the permitted distance from the center of a cluster. The results of unsupervised clustering are highly sensitive to initial parameters (eg, the starting points), are prone to bias and a lack of reproducibility, and cannot be readily validated even when different assumptions yield concordant findings.^59,60^ Latent class analysis uses a probability model to assign group membership and considers the complex relationships between multiple observed variables to define subgroups, but the approach suffers from a lack of standardization.^60,61^ Neural networks build on principal component analyses or topological data analyses to discern hidden patterns, but they carry the risk of overfitting, yielding a poor ability to make generalizations outside the training data. Most phenomapping studies in HFpEF have relied on unsupervised algorithmic clustering.^62^

All approaches to phenomapping represent a form of mathematical partitioning, which creates groupings of patients determined by certain dominant nonexclusive features. By definition, the analytical method coerces the identification of clusters even if they do not truly exist.^63^ Furthermore, because clusters are defined by central tendencies and not by external boundaries, they do not yield criteria that allow individual patients to be assigned to a specific phenogroup.^60,61^ The results of cluster analysis are dependent on the prevalence and distribution of measured variables, and it is exceptionally difficult to replicate the findings in 1 cohort in a second independent cohort of the same disease state. The reported number of identified clusters in different HFpEF studies has varied from 2 to 9.^62,64^ Achieving global maximum likelihood while fitting a model to observed data in 1 cohort limits the applicability of results to other datasets.

Although advocates of unsupervised cluster analysis regard the results as being data driven, the outputs of machine learning clustering are best characterized as being algorithm dependent. Results are determined by the initialization process that defines the proposed centers, and features such as scaling and distance metrics have a profound effect on the yield. Most biological variation is continuous, but unsupervised cluster analyses forces discreteness. In the end, clusters depend on the compactness of the data, which varies greatly from dataset to dataset.

Advocates of cluster analysis have written about its limitations. In a major review of phenotyping studies in 2023, Peters et al^62^ confirmed the lack of replicability of clustering analysis in cohorts of patients with HFpEF and concluded that phenomapping did not reliably yield implications for clinical care and clinical trials. Most identified clusters showed significant phenotypic overlap. For example, patients identified as members of a metabolic-obesity cluster are substantially similar to those in a natriuretic peptide deficiency cluster, but the two may be artificially distinguished if the analysis requires that a minimum number of clusters be identified. It is important to note that clusters can be formed only on the basis of available data, and to date, cluster analyses have not accounted for key variables relevant to HFpEF, for example, visceral adiposity, which drives both metabolic-obesity derangements and a natriuretic peptide deficiency.

It is inevitable that cluster analysis will yield groupings that carry prognostic significance because the clusters are based on central tendencies defined by variables that are already known to predict outcomes. The demonstration that clusters differ in outcomes does not validate the importance of the clusters; instead, it merely confirms that prognostic variables were included in the clusters. For example, it is known that older people and patients with chronic kidney disease or pulmonary hypertension with right ventricular dysfunction have a worse prognosis, whereas outcomes are more favorable in people with obesity (referred to as the obesity paradox). The prognosis of clusters is driven by the variables that are used to identify central tendencies. Although 1 study has claimed that phenomapping enhanced the ability to predict outcomes in HFpEF when added to models developed for HFrEF, the reported analyses demonstrated only marginal incremental discriminatory power.^3^ It is important to note that phenomapping has not been shown to yield c statistics that are meaningfully superior to simple biomarker-enhanced prognostic models specifically developed for HFpEF that are based on readily available clinical variables. In many instances, phenotypes may predict prognosis because they reflect different stages in the progression of HFpEF. Accordingly, patients frequently transition among phenogroups with the passage of time.^65^

Any potential value of phenomapping should be judged not by its capacity to prognosticate but by its ability to elucidate mechanisms of diseases or to identify differential responses to drugs that are known to be effective in the treatment of HFpEF.^60^ However, despite numerous efforts, this goal has not been achieved.^55,58,62,63^ Two reports of the utility of cluster analysis in predicting the magnitude of a treatment effect did not include any randomized control groups.^66,67^ In contrast, 6 reports pursued the possibility that phenomapping might identify responders to randomized treatment, with all 6 reports focusing on a single trial, the TOPCAT trial (Treatment of Preserved Cardiac Function Heart Failure With an Aldosterone Antagonist), yet each study yielded distinctly different results (Table 2).^68–73^ The 6 studies used diverse approaches of machine learning to identify as few as 2 and as many as 6 clusters, with each report describing a different set of phenogroups. Li et al^73^ applied the identical cluster analysis methodology across 3 cohorts of patients with HFpEF, but each cohort yielded a different set of phenogroups. In all 6 reports relevant to the TOPCAT trial, identified phenogroups differed in their prognosis, yet despite some signals discerned in within-group analyses, the magnitude of the response to spironolactone with respect to primary end-point events was not significantly different across any of the identified phenogroups (with all phenogroup-by-treatment interactions being not statistically significant).

**Table 2.**
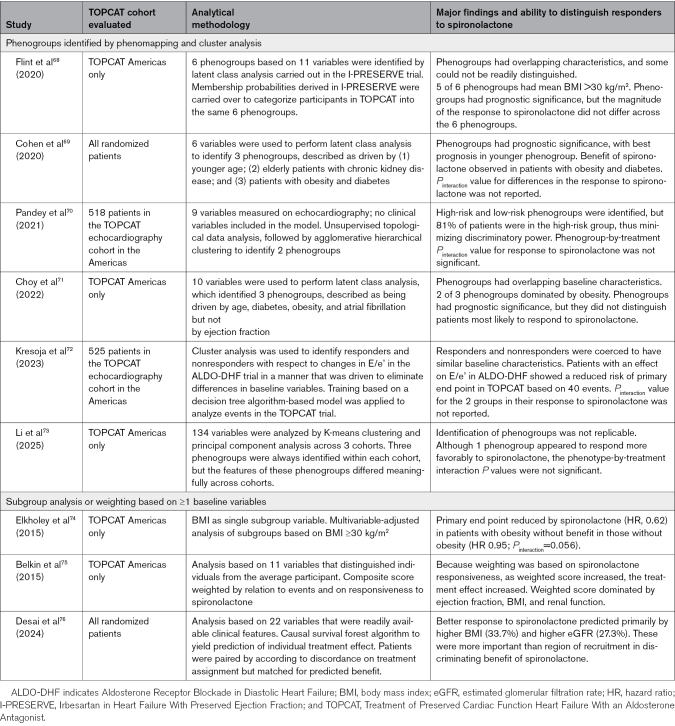
Studies of Phenotyping to Identify Responders to Spironolactone in the TOPCAT Trial

In contrast, relying on a single baseline variable, Elkholey et al^74^ identified patients with obesity as having an outsized benefit from spironolactone (body mass index–by–treatment interaction *P*=0.056), and body mass index was also identified as having a meaningful influence on the response to spironolactone by Belkin et al^75^ and Desai et al,^76^ who used weighed scores and causal survival forest algorithm to predict treatment effects. Obesity was also a determinant of the response to mineralocorticoid receptor antagonism in the FINEARTS-HF trial (Finerenone Trial to Investigate Efficacy and Safety Superior to Placebo in Patients With Heart Failure) with finerenone (treatment–by–body mass index interaction *P*=0.005).^77^ No study has demonstrated that phenomapping by cluster analysis is superior to the measurement of body mass index alone in identifying patients who respond favorably to mineralocorticoid receptor antagonism.

In the final analysis, phenomapping yields a descriptive taxonomy that is agnostic to a biological reality and is not positioned to yield falsifiable predictions.^60^ Admittedly, phenomapping provided a reassuring thought process when there were no broad-based effective treatments and no unifying hypothesis for HFpEF.^3^ However, recent advances in the field of HFpEF would seem to undermine the fundamental rationale for seeking to characterize phenotypic heterogeneity to understand the pathogenesis and to define the treatment of HFpEF in individual patients. It is well known that a single upstream mechanism can generate multiple downstream phenotypes. Accordingly, characterization of phenotypic heterogeneity by cluster analysis in HFrEF has not defined unique mechanisms or differential responses to treatments for HFrEF (which has a recognized unifying hypothesis and several broadly effective treatments; Figure 2).^1^

Similarly, despite a decade of effort, phenomapping has not identified a consistent set of phenogroups or defined a new disease state, and it has not been used in the design of clinical trials in HFpEF or in the identification of mechanistic targets for the development of new drugs. It is recognized that the HEARTSHARE initiative is performing multimodal phenotyping in HFpEF that includes clinical and pathophysiological features, imaging, and proteomics. However, these enormous datasets will be interrogated with unsupervised cluster analysis, machine learning, and phenomapping in the hope that previously reported phenogroups might be confirmed or that as-yet undiscovered phenotypic patterns might be discerned across multiple dimensions, although (as shown previously) this proposed analytical approach is demonstrably problematic.^60,78^

## Characterization of Effect-Modifying Variables in Positive HFpEF Trials

Large-scale trials with SGLT2 inhibitors (empagliflozin and dapagliflozin), mineralocorticoid receptor antagonists (finerenone), and GLP-1 receptor agonists (semaglutide and tirzepatide) have reported statistically significant and clinically meaningful benefits on their primary efficacy end points in analyses that were based on all randomized patients.^4–7,79,80^ In all these trials, although randomization was stratified on key variables to enhance discernment of potential heterogeneity, the magnitude of the treatment effect did not generally depend on variables assessed at baseline, including age, sex, presence or absence of coronary artery disease or diabetes, or baseline values for systolic blood pressure or renal function.^4–7,79,80^ In general, subgroup analyses of these trials revealed no replicable influence of a baseline variable on the direction or magnitude of the treatment effect observed in the overall trial. The finding of a favorable treatment effect across several classes of drugs in trials that were specifically designed to evaluate a broad population of patients with HFpEF, coupled with the general lack of observed heterogeneity of the effect size across numerous subgroups, is counter to the historical belief that undiscovered heterogeneity explained the disappointing results of randomized controlled HFpEF trials carried out before 2021.

However, it should be noted that the patents enrolled in large-scale trials of drugs for HFpEF were characterized by general uniformity with respect to the presence of excess adiposity. About 50% to 60% of patients in trials of SGLT2 inhibitors, mineralocorticoid receptor antagonists, and angiotensin receptor neprilysin inhibitors had a body mass index ≥30 kg/m^2^, and obesity was present in all patients in the trials with semaglutide and tirzepatide.^4–7,79,80^ More strikingly, central obesity (assessed by a waist-to-height ratio of ≥0.5) was present in >95% of patients with HFpEF enrolled across numerous large-scale trials,^81^ and excess visceral adiposity (measured by imaging) is evident in 85% of patients with HFpEF.^82^ A waist-to-height ratio of ≥0.5 has been shown to be consistently correlated with an expansion of visceral adipose tissue mass when assessed by noninvasive imaging.^83–85^

In patients with HFpEF, excess adiposity has been closely linked to more severe hemodynamic abnormalities, greater exercise intolerance, and a worse prognosis.^81,83,86–88^ The exceptional prevalence of adiposity in large-scale HFpEF trials is particularly impressive because, by protocol design, most large-scale HFpEF trials specifically excluded patients with a body mass index of >40 to 45 kg/m^2^; they also did not allow the enrollment of patients without marked increases in circulating natriuretic peptides at baseline, thereby excluding participants with marked obesity or excess visceral adiposity, 2 states that are characterized by natriuretic peptide suppression.

### Influence of Obesity on the Effect of HFpEF Drugs in Trials of Adiposity-Prevalent HFpEF

Does the degree of obesity or central adiposity influence the response to treatment with drugs with an efficacy in the management of HFpEF that has been demonstrated in large-scale randomized controlled clinical trials?

In the PARAGON-HF trial (Prospective Comparison of ARNI [Angiotensin Receptor–Neprilysin Inhibitor] With ARB Global Outcomes in Heart Failure With Preserved Ejection Fraction),^81^ patients with greater degrees of obesity had the largest decreases in the risk of cardiovascular death and hospitalizations for heart failure when treated with sacubitril/valsartan compared with valsartan. Patients with a body mass index ≥30 kg/m^2^ showed an ≈20% to 25% decrease in the risk of a major adverse heart failure outcome compared with minimal treatment effect in the patients with a body mass index of <30 kg/m^2^ (*P*_interaction_=0.06).

In the FINEARTS-HF trial,^77^ baseline values of body mass index were a significant linear determinant of the response to mineralocorticoid receptor antagonism with finerenone (*P*_interaction_=0.005). In patients with a body mass index of ≥30 kg/m^2^, treatment with finerenone decreased in the risk of cardiovascular death and hospitalizations for heart failure by ≈30% compared with minimal benefit in patients who were neither overweight nor obese. In addition, as noted, in patients with HFpEF who participated in the TOPCAT Americas trial, the presence of obesity identified patients who showed the greatest benefit with spironolactone.^74,75^

In the EMPEROR-Preserved trial (Empagliflozin Outcome Trial in Patients With Chronic Heart Failure With Preserved Ejection Fraction), patients with obesity appeared to show the most marked improvement in health status when treated with the SGLT2 inhibitor empagliflozin (*P*_interaction_=0.08).^89,90^ Changes in left ventricular filling pressure were correlated with changes in body weight in a randomized placebo-controlled trial of dapagliflozin.^91^

Unlike large-scale trials with other drugs, trials with drugs that signal through the GLP-1 receptor focused on patients with a body mass index ≥30 kg/m^2^, and, it is important to note, they did not exclude patients with marked obesity at baseline. In the SUMMIT trial, patients with the greatest severity of obesity at baseline and the greatest reduction in waist circumference after randomization showed the largest benefit on exercise tolerance when treated with tirzepatide (*P*=0.03 and *P*=0.0002, respectively).^92^ Of note, the severity of obesity was a stratification variable for randomization in the trial.

At the same time, the presence of obesity does not identify responders in multicenter controlled clinical trials that have reported neutral results and that have evaluated interventions that are not linked to an action to normalize adipokine biology.^93,94^

These findings support the premise that the magnitude of excess adiposity may be an important effect modifier in patients with HFpEF. The more pronounced effect of HFpEF drugs in patients with a body mass index ≥30 kg/m^2^ raises the intriguing hypothesis that large-scale positive trials might not have demonstrated favorable effects on key efficacy variables if they had focused only on individuals with HFpEF mimics, who typically do not have excess adiposity. Conversely, if the trials had permitted the participation of patients with class III obesity or with lower natriuretic peptide levels at baseline, the magnitude of the observed treatment effects in these trials might have been larger than reported.

These results are consistent with the hypothesis that obesity and visceral adiposity have mechanistic importance in the pathogenesis of HFpEF.^32^ The American Heart Association has formally recognized excess and dysfunctional visceral fat as the upstream root cause of HFpEF, which is described as an advanced stage of the cardiovascular-kidney-metabolic syndrome.^95^ Obesity and visceral adiposity are the principal risk factors for the development of HFpEF in the general population,^96,97^ and it has been hypothesized that the expansion of visceral fat mass leads to a shift in its secretory profile, which is dominated by a cytoprotective suite of molecules in lean healthy people but is transformed into to a proinflammatory and prohypertrophic secretory pattern in patients with excess adiposity.^32^ In experimental models, a shift in the secretion of these adipokines is sufficient to cause the structural and functional features of HFpEF in experimental models, as evidenced by studies demonstrating that adipose tissue–specific silencing of deleterious adipokines suppresses their circulating levels in the bloodstream, resulting in attenuation of their effect on the myocardium and amelioration of HFpEF.^32^ SGLT2 inhibitors, mineralocorticoid receptor antagonists, and GLP-1 receptor agonists act to shrink visceral adipose tissue depots and normalize the deleterious adipokine imbalance, and these actions may contribute meaningfully to their benefits in HFpEF.^32^ A pathogenetic shift in the adipokine profile in patients with excess adiposity is consistent with findings that the baseline presence and severity of obesity represent the only meaningful effect modifier that has been observed in large-scale HFpEF trials that have demonstrated a favorable effect of treatment.

### Representativeness of Participants in Large-Scale HFpEF Trials

Is the general uniformity with respect to central adiposity in large-scale trials of HFpEF representative of HFpEF in the general community? In the 1980s, physicians believed that uncontrolled hypertension in an aging population was the principal driver of the development of a hypertrophic cardiomyopathy that could lead to HFpEF. However, with the emergence and acceleration of an epidemic of obesity, the primary driver of HFpEF in the clinical community in the current era is likely related to an expansion of fat mass. This exceptional prevalence is true even in rural community dwellers in Asia.^98^

Nevertheless, it is possible that hypertension in aging people may represent the cause of HFpEF in a proportion of patients seen in clinical practice but that eligibility criteria diminished the participation of such patients in large-scale trials. The criteria for participation in large-scale trials of patients with HFpEF specifically excluded the participation of patients with severe uncontrolled hypertension or advanced chronic kidney disease, but as noted, they also simultaneously diminished the participation of patients with severe obesity. Clinical trials appropriately excluded the enrollment of patients with HFpEF mimics (eg, hypertrophic or infiltrative cardiomyopathy, cardiac amyloidosis, valvular heart failure, pericardial disease, and end-stage kidney disease), which require specialized treatment strategies. Because of these eligibility criteria, most of what we know about the benefits of angiotensin receptor neprilysin inhibitors, SGLT2 inhibitors, mineralocorticoid receptor antagonists, and GLP-1 receptor agonists in patients with HFpEF is determined by populations in whom the disease may be driven primarily by central obesity and excess visceral adiposity.

## Summary and Conclusions

The available evidence suggests that the patients enrolled in large-scale randomized clinical trials that demonstrated a favorable treatment effect in patients with HFpEF were primarily patients with central obesity and that the magnitude of the benefit was related to the degree of adiposity, although the trials excluded the participation of patients with the most marked obesity and adiposity-related suppression of natriuretic peptides. No other baseline variable in these positive-outcome trials influenced the presence or magnitude of the treatment effect. It seems likely that, if patients with marked obesity or visceral adiposity had been included, the trials that demonstrated a broad-based treatment effect with angiotensin receptor neprilysin inhibitors, SGLT2 inhibitors, mineralocorticoid receptor antagonists, and GLP-1 receptor agonist might have demonstrated a larger treatment effect.

At the same time, the belief that characterization of the phenotypic heterogeneity of patients with HFpEF reveals mechanistic insights that can lead to the development of novel treatments, discern new disease states, or identify patients who selectively benefit from an established therapy is based largely on post hoc subgroup analyses of neutral HFpEF trials. Subgroup findings in neutral trials of drugs for heart failure have historically failed to yield a replicable result, and more complex assessments involving invasive hemodynamic measurements, imaging, or proteomics have not borne fruit. Although cluster analysis and machine learning seek to combine phenotypic features across multiple domains, they have not yielded phenogroups that can be replicated, be identified by clinicians in the clinical setting, distinguish responders from nonresponders in clinical trials, or reveal novel causal mechanisms, new treatments, or undiscovered diseases. Accordingly, these approaches have not been used prospectively for the design, execution, or analysis of randomized trials of any novel therapeutic intervention, and they have not advanced a single new therapeutic intervention for HFpEF over the past decade.

Despite assumptions about the existence of exceptional mechanistic heterogeneity in HFpEF, large-scale trials carried out over the past 10 to 15 years have enrolled a broad-based population of patients who have demonstrated a remarkable consistency of treatment responses across clinically relevant subgroups. Rather than supporting any need to characterize exceptional phenotypic complexity, simple measurements of adiposity appear to identify treatment responders among effective drugs for HFpEF and are consistent with current mechanistic frameworks to explain the pathogenesis of HFpEF in most people with the disease in the current era.

These observations should not be construed to imply that therapeutic interventions that are directed at downstream derangements might not be worthwhile. Although neurohormonal activation represents a unifying mechanism for HFrEF, patients with HFrEF who have severe mitral regurgitation without marked left ventricular enlargement may nevertheless benefit from transcatheter mitral valve repair. Similarly, although visceral adiposity promotes both hypertension and atrial fibrillation,^99,100^ it is possible that control of these downstream exacerbating conditions with the use of antihypertensive treatments and catheter ablation of atrial arrhythmias might still be beneficial in the management of HFpEF,^100^ and ongoing trials are poised to test these hypotheses. Such mechanisms may also be valid targets in the management of HFrEF.

However, most efforts to characterize phenotypic heterogeneity in HFpEF to date have not been directed at simple, clinically available variables to identify these downstream events. Instead, they have assumed that complex multimodal interrogation of phenotypic diversity is required to reveal causal mechanistic upstream pathways, new potential disease states, and differential treatment responses based on variables that are not readily assessed in clinical practice, an assumption that differs fundamentally from our current conceptualization of HFrEF. The possibility that deep or complex phenotyping for HFpEF can reveal undiscovered causal mechanisms or optimize the utilization of broad-based treatments represents a conceptual framework that has not been validated, is not well supported, and may no longer be needed.

## Article Information

### Disclosures

Dr Packer has received consulting fees from AbbVie, Actavis, Alnylam, Altimmune, Ardelyx, Amgen, ARMGO, AstraZeneca, Attralus, Biopeutics, Boehringer Ingelheim, Caladrius, Casana, CSL Behring, Cytokinetics, Daiichi-Sankyo, Eli Lilly and Company, Imara, Medtronic, Moderna, Novartis, Pharmacosmos, Preload, PriveBio, Regeneron, and Salamandra. Dr Petrie has received research funding form Boehringer Ingelheim, Roche, SQ Innovations, AstraZeneca, Novartis, Novo Nordisk, Medtronic, Boston Scientific, and Pharmacosmos; and has consulted or served on committees for Abbott, Akero, Applied Therapeutics, Amgen, AnaCardio, Biosensors, Boehringer Ingelheim, Corteria, Novartis, AstraZeneca, Novo Nordisk, Abbvie, Bayer, Horizon Therapeutics, Foundry, Takeda, Cardiorentis, Pharmacosmos, Siemens, Eli Lilly, Vifor, New Amsterdam, Moderna, Teikoku, LIB Therapeutics, 3R Lifesciences, Reprieve, FIRE 1, Corvia, and Regeneron. Dr Butler has consulting relationships with Abbott, American Regent, Amgen, Applied Therapeutic, AskBio, Astellas, AstraZeneca, Bayer, Boehringer Ingelheim, Boston Scientific, Bristol Myers Squibb, Cardiac Dimension, CardioCell, Cardior, CSL Bearing, CVRx, Cytokinetics, Daxor, Edwards, Element Science, Faraday, Foundry, G3P, Innolife, Impulse Dynamics, Imbria, Inventiva, Ionis, Levator, Lexicon, Lilly, LivaNova, Janssen, Medtronic, Merck, Occlutech, Owkin, Novartis, Novo Nordisk, Pfizer, Pharmacosmos, Pharmain, Prolaio, Pulnovo, Regeneron, Renibus, Roche, Salamandra, Salubris, Sanofi, SC Pharma, Secretome, Sequana, SQ Innovation, Tenex, Tricog, Ultromics, Vifor, and Zoll. Dr Zannad has consulting relationships with Alnylam, Bayer, Biopeutics, Boehringer, CellProthera, Cereno, Centrix, Corteria, CVRx, CVCT, Lilly, Lupin, Merck, Novo Nordisk, Opalia Recordati, Owkin, Polygon, Ribocure, Riche, and Viatri. Dr Zannad is on steering committees and data and safety monitoring and advisory boards for Alnylam, Bayer, Biopeutics, Boehringer, CellProthera, Cereno, Corteria, CVRx, Merck, Owkin, Ribocure, and Roche; owns equities and stock options in Polygon, Cereno Pharmaceutical, and CVCT; and is on the Speakers Bureau for Bayer, Boehringer, Centrix, CVRx, Lupin, Opalia Recordati, Merck, Novo Nordisk, and Viatris. Dr Lam has received support from Novo Nordisk and Roche Diagnostics; has served as consultant or on the advisory board/steering committee/executive committee for Alnylam Pharma, AnaCardio AB, Applied Therapeutics, AstraZeneca, Bayer, Biopeutics, Boehringer Ingelheim, Boston Scientific, Bristol Myers Squibb, Corteria, CPC Clinical Research, Cytokinetics, Eli Lilly, Impulse Dynamics, Intellia Therapeutics, Ionis Pharmaceutical, Janssen Research & Development LLC, Medscape/WebMD Global LLC, Merck, Novartis, Novo Nordisk, Quidel Corporation, Radcliffe Group Ltd, Roche, and Us2.ai; and is the cofounder and nonexecutive director of Us2.ai. Dr Vaduganathan has received research grant support from, served on advisory boards for, or had speaker engagements with Alnylam Pharmaceuticals, American Regent, Amgen, AstraZeneca, Bayer AG, Baxter Healthcare, BMS, Boehringer Ingelheim, Chiesi, Cytokinetics, Esperion, Fresenius Medical Care, Idorsia Pharmaceuticals, Lexicon Pharmaceuticals, Merck, Milestone Pharmaceuticals, Novartis, Novo Nordisk, Pharmacosmos, Recordati, Relypsa, Roche Diagnostics, Sanofi, and Tricog Health; Dr Vaduganathan participates on clinical trial committees for studies sponsored by Amgen, AstraZeneca, Galmed, Novartis, Bayer AG, Occlutech, Pharmacosmos, and Impulse Dynamics. Dr Borlaug receives research support from the National Institutes of Health and the US Department of Defense, as well as research grant funding from AstraZeneca, Axon, Corvia, Novo Nordisk, and Tenax Therapeutics; has served as a consultant for Actelion, Amgen, Aria, Axon Therapies, BD, Boehringer Ingelheim, Cytokinetics, Edwards Lifesciences, Lilly, Imbria, Janssen, Merck, Novo Nordisk, NGM, NXT, and VADovations; and is named inventor (U.S. Patent No. 10,307,179) for the tools and approach for a minimally invasive pericardial modification procedure to treat heart failure. Dr Burkhoff has received equity from Harvi Dynamics Inc and has received an unrestricted educational grant from Abiomed Inc and consulting fees from Boston Scientific, Medtronic, and TherOx. The other authors report no conflicts of interest.
